# Trastornos linfoproliferativos en una cohorte de pacientes adultos con trasplante hepático atendidos en un hospital de referencia en Bogotá, Colombia

**DOI:** 10.7705/biomedica.4861

**Published:** 2020-06-30

**Authors:** Leonardo F. Jurado, Andrés Gómez-Aldana, Mónica Tapias, Daniela Cáceres, Alonso Vera, Rocío del Pilar López-Panqueva, Rafael E. Andrade

**Affiliations:** 1 Departamento de Patología y Laboratorios, Hospital Universitario Fundación Santa Fe de Bogotá, Bogotá, D.C., Colombia Hospital Universitario Fundación Santa Fe de Bogotá BogotáD.C Colombia; 2 Departamento de Patología, Facultad de Medicina, Universidad Nacional de Colombia, Bogotá, D.C., Colombia Universidad Nacional de Colombia Facultad de Medicina Universidad Nacional de Colombia BogotáD.C Colombia; 3 Pathology Department, McGill University, Montreal, Quebec, Canada McGill University McGill University MontrealQuebec Canada; 4 Servicio de Trasplante Hepático, Hospital Universitario Fundación Santa Fe de Bogotá, Bogotá, D.C., Colombia Hospital Universitario Fundación Santa Fe de Bogotá BogotáD.C Colombia; 5 Facultad de Medicina, Universidad de los Andes, Bogotá, D. C., Colombia Universidad de los Andes Facultad de Medicina Universidad de los Andes BogotáD. C Colombia

**Keywords:** trastornos linfoproliferativos, linfoma, trasplante de hígado, Colombia, Lymphoproliferative disorders, lymphoma, liver transplantation, Colombia

## Abstract

**Introducción.:**

Los trastornos linfoproliferativos después de un trasplante se caracterizan por la proliferación descontrolada de linfocitos como consecuencia del tratamiento inmunosupresor posterior a este.

**Objetivo.:**

Caracterizar clínica y patológicamente los casos de trastornos linfoproliferativos después de trasplante (*Post-Transplant Lymphoproliferative Disorders*, PTLD) en una cohorte de pacientes adultos con trasplante de hígado atendidos a lo largo de 15 años en el Hospital Universitario Fundación Santa Fe de Bogotá.

**Materiales y métodos.:**

Se hizo un estudio observacional retrospectivo a partir de la revisión de las bases de datos de la Unidad de Trasplante Hepático y del Departamento de Patología del Hospital en busca de los casos de PTLD diagnosticados durante el periodo de estudio. Se recolectó la información epidemiológica, clínica y patológica, y se adelantaron los análisis estadísticos.

**Resultados.:**

Durante el periodo de estudio, hubo 572 pacientes con trasplante de hígado, la incidencia de trastornos linfoproliferativos fue de 2,44 %, el 79 % en hombres, y la edad promedio en el momento del diagnóstico fue de 62,5 años. El 71 % de los casos se presentó durante los primeros 12 meses después del trasplante y el mismo porcentaje fue seropositivo para el virus de Epstein-Barr (EBV). El fenotipo patológico más frecuente fue el monomorfo y la mayoría de los tumores se detectaron en el hilio hepático. La supervivencia al año fue del 50 %.

**Conclusiones.:**

Llamó la atención el alto porcentaje de casos de presentación temprana, así como la gran frecuencia de seropositividad para el EBV tanto en los donantes como en los receptores. Deben adelantarse estudios más detallados para una mejor comprensión de esta enfermedad en el país. Este es el primer análisis clínico y patológico de PTLD en pacientes con trasplante de hígado adelantado en Colombia hasta la fecha.

## Abstract (en)

**Introduction:**

The post-transplant lymphoproliferative disorders (PTLD) are characterized by an uncontrolled pathological lymphoid proliferation as a consequence of transplant immunosuppression therapy.

**Objective:**

To characterize the clinical and pathological characteristics of PTLD in a cohort of adult patients with liver transplant during a 15 year period at the *Hospital Universitario Fundación Santa Fe de Bogota*.

**Materials and methods:**

We conducted an observational retrospective study by searching for the PTLD cases diagnosed during the study period in the databases of the Liver Transplantation Unit and the Pathology Department. We collected the epidemiological, clinical, and pathological information and performed the corresponding statistics analyses.

**Results:**

During the research period, 572 patients were transplanted; the incidence of PTDL was 2.44%; 79% of them were man and the average age at the time of diagnosis was 62.5 years; 71% of the cases were diagnosed during the first year after the transplant and the same percentage EBV-seropositive patients. The most frequent pathological phenotype was monomorphic and the majority of tumors was detected in the hepatic hilum. The one-year survival was 50%.

**Conclusion:**

The high proportion of early cases and the high frequency of Epstein-Barr virus seropositivity both in donors and receptors drewour attention. More studies are necessary to have a better understanding of this condition in Colombia. This is the first PTLD clinical and pathological analysis in liver-transplant patients from Colombia to date.

El trasplante de hígado se ha convertido en una opción terapéutica capaz de salvar la vida de pacientes con falla hepática aguda, enfermedad hepática terminal o neoplasias hepáticas primarias. Sin embargo, el tratamiento inmunosupresor crónico para reducir el riesgo de rechazo del injerto aumenta la incidencia de varias condiciones, incluidas neoplasias como los trastornos linfoproliferativos después de trasplante (*Post-Transplant Lymphoproliferative Disorders*, PTLD) ^1^. Los primeros reportes de PTLD se publicaron en 1968 ^2^ y el término se acuñó en 1984 ^3^.

Los PTLD se caracterizan por una proliferación anormal de linfocitos en el contexto de la inmunosupresión extrínseca posterior al trasplante de un órgano ^4^^,^^5^. Se puede asociar con los trasplantes de órgano sólido (*Solid Organ Transplant,* SOT) o con los de progenitores hematopoyéticos (*Haemathopoyetic Stem Cell Transplant*, HSCT). Dado que en los primeros las condiciones resultantes son diferentes a las que se presentan en los segundos, es usual referirse a estas de forma independiente como SOT-PTLD y HSCT-PTLD ^5^.

Los PTLD corresponden a una complicación grave de los dos tipos de trasplante. Desde el punto de vista patológico, estos trastornos pueden incluir desde una reacción tisular similar a la de una infección hasta un linfoma; se sabe que alrededor del 70 % de los casos se relaciona con la infección por el virus de Epstein-Barr (EBV) ^1^^,^^5^.

Entre los factores de riesgo más importantes para el desarrollo de un PTLD, están el estado de infección por el EBV en el momento del trasplante, el órgano trasplantado, y el tipo y la duración del esquema inmunosupresor. La reconstitución del sistema inmunitario mediante la reducción o suspensión del tratamiento inmunosuporesor se considera el eje fundamental del manejo, aunque en una gran proporción de pacientes se requieren estrategias adicionales ^1^^,^^4^^,^^5^.

Se estima que la infección por el EBV es la responsable principal de esta condición al inducir la proliferación anormal de linfocitos en 50 a 80 % de los PTLD, especialmente en los casos de inicio temprano (menos de dos años después del trasplante). En el porcentaje restante de casos (20 a 50%) que, además, son negativos para el EBV, se desconoce la causa desencadenante específica ^5^^,^^6^.

Los receptores de órganos sólidos tienen aproximadamente 10 veces más riesgo de desarrollar linfoma que la población general ^7^ y la incidencia de PTLD en ellos es de cerca del 20 %; sin embargo, varía según el órgano trasplantado. Dicha diferencia puede estar relacionada con la cantidad de tejido linfoide trasplantado y la intensidad de la inmunosupresión requerida para prevenir el rechazo agudo del injerto, lo que depende del órgano ^8^. Los receptores de trasplante intestinal o de más de un órgano, tienen el más alto riesgo (12 a 17 %), seguidos de los receptores de pulmón (6 a 10 %), corazón (3 a 5 %), hígado (2 a 3 %) y riñón (1,5 a 2,5 %) ^9^.

Inicialmente, el trasplante de hígado se asociaba con un alto riesgo de PTLD comparado con el de otros órganos ^10^, pero dicho riesgo ha disminuido debido a la tendencia a disminuir o, incluso, suspender el tratamiento inmunosupresor después del trasplante ^11^^,^^12^. En los niños con trasplante de hígado, la incidencia de PTLD también ha disminuido debido a la modulación preventiva del tratamiento inmunosupresor, así como la valoración sistemática de la carga viral del EBV ^8^^,^^13^.

En su amplio estudio retrospectivo, Opelz, *et al.*^4^, observaron un riesgo relativo de linfoma no Hodgkin a los cinco años del trasplante de hígado de 29,9; el riesgo relativo más alto fue el de trasplante de pulmón y corazón, seguido por el de pulmón, corazón, hígado, páncreas y riñón con donante cadavérico. Este aumento del riesgo en trasplantes de cualquiera de los órganos se hizo más evidente en la población pediátrica, resaltando la influencia del alto porcentaje de seronegatividad en los niños.

En otro estudio retrospectivo, se analizaron 140 especímenes de biopsia de pacientes con PTLD recolectados durante 20 años y los hallazgos confirmaron las diferencias del riesgo según el órgano ^1^, observándose el más alto en los receptores de corazón (5,0 %), seguido de los de pulmón (3,2 %), hígado (2,8 %), células madre hematopoyéticas (1,7 %) y riñón (1,5 %), con una incidencia promedio en toda la población con trasplante de 2,12 %.

En una gran serie de 4.000 casos de trasplante de hígado, se observó una incidencia de PTLD de 4,3 %, con una clara diferencia entre niños (9,7 %) y adultos (2,9 %) ^14^.

En este estudio se describieron y analizaron las características clínicas y los hallazgos patológicos de los pacientes adultos con trasplante de hígado y trastorno linfoproliferativo posterior a este, atendidos en el Hospital Universitario Fundación Santa Fe de Bogotá a lo largo de 15 años.

## Materiales y métodos

Se revisaron las bases de datos de la Unidad de Trasplante Hepático del Hospital correspondientes al periodo comprendido entre enero de 2002 y diciembre de 2017. Se buscaron las historias clínicas de los pacientes mayores de 18 años que desarrollaron PTLD y se extrajo la información demográfica básica (sexo, edad), y sobre los antecedentes clínicos relevantes (enfermedad hepática primaria, tiempo transcurrido desde el trasplante de hígado hasta el diagnóstico de PTLD, estado de infección por HCV), esquema de inmunosupresión, estado clínico y paraclínico al momento del diagnóstico (fiebre, dolor abdominal, ictericia, alteración del perfil hepático), tratamiento instaurado una vez se hizo el diagnóstico del PTLD, localización anatómica de la lesión neoplásica, condiciones conocidas como factores de riesgo para PTLD (antecedente de rechazo celular agudo, recaída de infección por HCV, estado serológico para EBV en el donante y el receptor) y muerte durante el periodo de seguimiento.

Se revisaron los reportes de los estudios de patología de cada paciente y se recolectó la información referente a la clasificación del PTLD según la Organización Mundial de la Salud (OMS) del 2016 ^15^, así como sobre los PTLD monomorfos, la positividad de los marcadores de inmunohistoquímica (IHQ) para EBV, citomegalovirus (CMV) y antígeno CD20, el índice de proliferación Ki 67, la detección de EBV por hibridación *in situ* y la proteína latente de membrana por IHQ, y, además, la presencia de compromiso tumoral en la médula ósea.

### Consideraciones éticas

El presente trabajo contó con la aprobación del comité institucional de ética en investigación.

## Resultados

Durante el periodo de estudio, se encontraron 572 pacientes con trasplante de hígado. Hubo 14 pacientes con diagnóstico de trastorno linfoproliferativo, la gran mayoría (79 %) de los cuales eran hombres, en tanto que la edad promedio fue de 62,5 años (desviación estándar, DE=3,55) (cuadro 1).

**Cuadro 1:** Características generales de los pacientes, antecedentes y forma de presentación

Variable	n (n=14)	%
Edad promedio (años) (Rango: 55 a 67)	62,5	
Sexo		
Hombres	11	78,57
Mujeres	3	
Aparición del PTLD		
Temprana (<12 meses)	10	71,42
Tardía (>12 meses)	4	28,57
Enfermedad hepática primaria		
Cirrosis alcohólica	4	28,57
Esteatohepatitis no alcohólica	3	21,43
Cirrosis biliar primaria	2	14,29
Infección por HCV y hepatocarcinoma	2	14,29
Infección por HCV	1	7,14
Cirrosis biliar primaria más superposición	1	7,14
Sobrecarga de hierro	1	7,14
Esquema de inmunosupresión		
Ciclosporina más micofenolato	10	71,43
Tacrolimus más micofenolato	2	14,29
Tacrolimus	1	7,14
Sirolimus	1	7,14
Forma de presentación		
Alteración del perfil hepático	8	57,14
Dolor abdominal	3	21,43
Fiebre	2	14,29
Ictericia	1	7,14
Asintomático	1	7,14
Tratamiento ofrecido		
Rituximab	13	92,8

HCV: virus de la hepatitis C

El promedio del tiempo transcurrido entre el trasplante y el diagnóstico del PTLD fue de 18,4 meses (rango: 2 a 72). Con un tiempo de hasta 12 meses antes de la aparición, se consideró de inicio temprano y, con más de 12 meses, de inicio tardío. En 71 % de los casos, fue de inicio temprano (cuadro 1). El 42 % de los pacientes falleció durante el primer año de seguimiento y, el 50 %, a lo largo del periodo de estudio.

La enfermedad hepática primaria más frecuente fue la cirrosis alcohólica (28,57 %), seguida por la esteatohepatitis no alcohólica (21,43 %). El esquema de inmunosupresión más frecuentemente utilizado (71,43 %) fue la combinación de ciclosporina con micofenolato. En cuanto a la presentación clínica, solo tres pacientes presentaron dolor abdominal, y la fiebre y la ictericia fueron incluso menos frecuentes, en tanto que, en 8 (57,14%) pacientes, se encontraron alteraciones del perfil hepático (cuadro 1).

En cuanto a la frecuencia de factores de riesgo para PTLD, cinco pacientes tenían antecedentes de rechazo celular agudo y en tres hubo recaída virológica de la infección por HCV. Por otro lado, según los estudios previos al trasplante, seis de los donantes y diez de los receptores eran positivos para EBV (cuadro 2).

**Cuadro 2:** Frecuencia de factores de riesgo para PTLD

Variable	n (n=14)	%
Rechazo celular agudo	5	35,71
Recaída virológica de HCV	3	21,43
Estado serológico de EBV en el donante		
Positivo	6	42,86
Negativo	5	35,71
Sin dato	3	21,43
Estado serológico de EBV en el receptor		
Positivo	10	71,43
Negativo	1	7,14
Sin dato	3	21,43

HCV: virus de la hepatitis C; EBV: virus de Epstein-Barr

**Figura 1:**
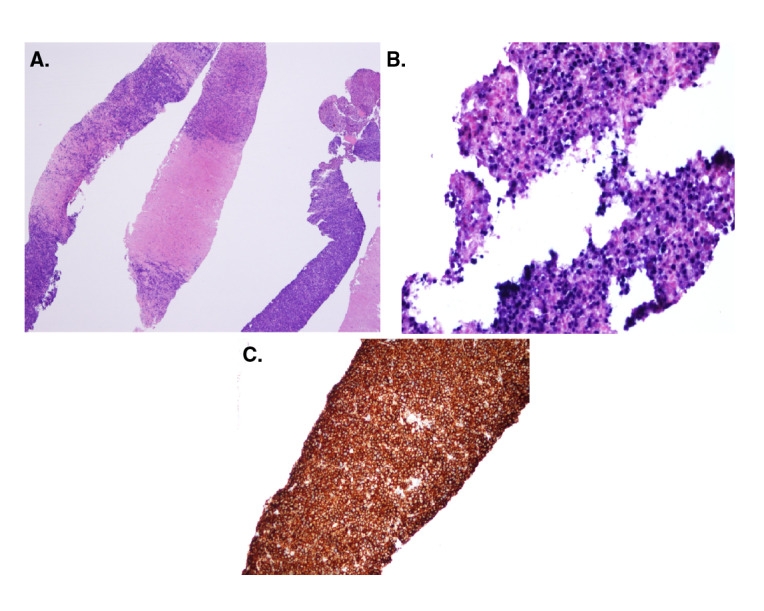
Plasmocitoma. **A)** Infiltrado tumoral constituido por abundantes plasmocitos. Hematoxilina-eosina, 40X. **B)** Virus de Epstein-Barr, positivo. **C)** Inmunohistoquímica: CD138, positivo, 40X

Con respecto a los hallazgos del estudio patológico del espécimen tumoral y los estudios complementarios, según el esquema de clasificación histológica de la OMS ^15^, la gran mayoría (71,42 %) se clasificó como monomorfo, y el subtipo más frecuente (54,54 %) fue el linfoma B difuso de célula grande, en tanto que la localización más frecuente de la neoplasia fue el hilio hepático, con 10 casos; hubo un caso de plasmocitoma y uno de linfoma de Burkitt (figuras 1 y 2). En cuanto al compromiso extrahepático, se encontró un caso con infiltración de la médula ósea (cuadro 3).

**Figura 2:**
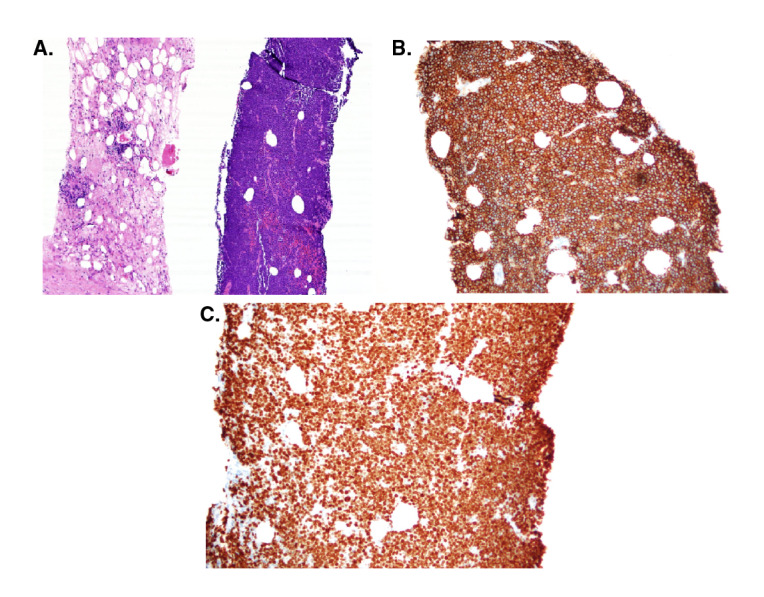
Linfoma de Burkitt. **A)** Infiltrado tumoral. Hematoxilina-eosina, 10X. **B)** Inmunohistoquímica: CD20, 20X. **C)** Inmunohistoquímica: Ki67,20X

**Cuadro 3:** Clasificación histológica y otros hallazgos

Variable	n (n=14)	%
Clasificación del PTLD según la OMS		
Monomorfo	10	71,42
Polimorfo	2	14,29
Linfoma de Hodgkin clásico	1	7,14
Lesión temprana	1	7,14
Subclasificación de PTLD monomórfico	(n=10)	100
Linfoma B difuso de célula grande	6	54,54
Plasmocitoma	1	9,09
Linfoma de Burkitt	1	9,09
Otros	2	18,18
Localización de la lesión		
Hilio hepático	10	57,14
Hígado	3	35,71
Bazo	1	7,14
Compromiso de la médula ósea	1	7,14
Otros hallazgos		
CD20 por IHQ, positivo	13	92,9
EBV por IHQ, positivo	12	85,71
CMV (por IHQ o PCR en suero), positivo	3	21,43
Índice de proliferación Ki 67	Promedio 54,6	DE 18,9
Modo de detección de EBV		
Hibridación in situ	9	64,28
Detección de LMP1 por IHQ	2	14,29
Hibridación in situ más LMP1 por IHQ	3	21,43

EBV: virus de Epstein-Barr; CMV: citomegalovirus; IHQ: inmunohistoquímica; PCR: *Polymerase Chain Reaction*; LMP1: *Latent Membrane Protein* 1; DE: desviación estándar

## Discusión

Durante los 15 años analizados, 572 pacientes se sometieron a trasplante de hígado en la institución y, de ellos, 14 fueron diagnosticados con PTLD, es decir que la frecuencia de PTLD en la cohorte estudiada fue de 2,44 %, hallazgo muy similar al reportado por diversos estudios en otros países. En Estados Unidos, Jain,*et al*. ^14^, en 4.000 pacientes con trasplante de hígado y un periodo de seguimiento de 20 años, registraron una frecuencia de 2,9 % en pacientes adultos. Mendizábal, *et al.*^16^, en 1.621 pacientes de Argentina, Chile y Brasil, reportaron una frecuencia de PTLD de 1,7 %.

Casi el 80 % de los pacientes del presente estudio correspondía a hombres y la edad promedio fue de 62,5 años; por otro lado, en los estudios de Mumtaz, *et al.*^17^, Kremers, *et al.*^18^, y Mendizábal, *et al.*^16^, el 43, el 44 y el 62 % correspondieron a mujeres, respectivamente, en tanto que la edad promedio de presentación fue comparable, aunque se observó una gran diferencia en el rango, con pacientes desde los 18 hasta los 75 años.

En cuanto al tiempo transcurrido entre el trasplante de hígado y el diagnóstico del PTLD, la gran mayoría (71,42 %) de los pacientes del presente estudio fue diagnosticada antes de cumplirse los 12 meses del trasplante (entre 2 y 11 meses) y, además, el 70 % de ellos eran positivos para el EBV, lo que se ha asociado con el desarrollo temprano de PTLD ^1^^,^^5^. Este hallazgo contrasta con lo informado por Mendizábal, *et al.*^16^, y Mumtaz, *et al.*^17^, quienes reportaron frecuencias de aparición temprana muy inferiores, de 27 y 28 %, respectivamente. Las indicaciones para el trasplante de hígado en los pacientes incluidos en el presente estudio, se enumeran en el cuadro 1, información que varía mucho entre los diversos estudios.

Es bien sabido que la administración permanente del tratamiento inmunosupresor es un importante factor de riesgo para PTLD. Debido a que la mayoría de protocolos de trasplante incluye esquemas combinados de terapia de inducción y de mantenimiento, es difícil determinar el impacto de cada medicamento por separado. Sin embargo, aunque es objeto de controversia, algunos agentes parecen estar directamente asociados con el desarrollo de PTLD, en tanto que otros pueden considerarse, incluso, protectores ^1^^,^^5^^,^^10^.

En varios estudios se ha demostrado que los inhibidores de la calcineurina (ciclosporina, tacrolimus) se asocian con un mayor riesgo de desarrollar PTLD. Dadas las fuertes propiedades inmunosupresoras del tacrolimus, este agente parece asociarse con un mayor riesgo comparado con la ciclosporina en distintos órganos, incluido el hígado. Por el contrario, el antimetabolito micofenolato de mofetilo aparentemente no se asocia con aumento de riesgo de PTLD, lo que también se ha observado en el trasplante de órganos diferentes al hígado ^1^^,^^5^.

Sin embargo, el esquema de inmunosupresión más frecuentemente utilizado (71,43 %) en la serie aquí descrita fue la combinación de ciclosporina y micofenolato, seguida de tacrolimus y micofenolato (14,29 %). En varios estudios, incluidos los llevados a cabo en Latinoamérica ^10^^,^^16^, se evidencia que estos medicamentos también han sido los más frecuentemente empleados como inmunosupresores, aunque en porcentajes un poco inferiores, ya que se han incorporado otros como parte del arsenal farmacológico.

Por otro lado, en varios estudios se ha demostrado un mayor riesgo de sufrir episodios de rechazo celular agudo en los pacientes con trasplante de hígado, lo que se atribuye al agresivo tratamiento inmunosupresor que debe administrarse ^5^^,^^10^. En la presente serie, cinco (35,7 %) pacientes experimentaron por lo menos un episodio de rechazo celular agudo antes del diagnóstico del PTLD, lo que contrasta levemente con los hallazgos de otros estudios que han reportado una frecuencia de dicho rechazo de hasta el 54 % ^16^.

Se reconoce que el ser seronegativo para el EBV en el momento del trasplante aumenta el riesgo de desarrollar PTLD en más de 12 veces, en comparación con el riesgo de receptores que ya son seropositivos ^7^. El riesgo más alto les corresponde a quienes se infectan justo después de la recepción del injerto, probablemente como consecuencia de recibir el injerto de un donante positivo para EBV ^7^. Asimismo, se sabe que la infección por EBV se relaciona con el riesgo de desarrollar tempranamente el PTLD (menos de 12 meses después de trasplante). En el presente estudio, el 43 % de los donantes y el 71 % de los receptores eran seropositivos para EBV, lo que se relaciona, además, con la gran frecuencia (más del 70 %) de PTLD de inicio temprano.

En cuanto a la clasificación patológica del PTLD según el esquema de la OMS ^15^, la gran mayoría fue monomorfo (71,42 %) y, un porcentaje menor, fue polimorfo (14,29 %); un caso se clasificó como linfoma de Hodgkin clásico y otro como lesión temprana (7,14 %). Entre los tumores monomorfor, el subtipo más frecuente fue el linfoma B difuso de célula grande (cuadro 3). Estos hallazgos concuerdan con lo descrito en la literatura, por ejemplo, en un estudio en Hong Kong ^19^, se reportó una frecuencia de 73 % de tumores monomórficos, en tanto que, en otro realizado en Estados Unidos ^18^, se informó una frecuencia de 85 %, y en el estudio ya comentado que incluyó pacientes latinoamericanos, se reportó una frecuencia de 63 % de tumores monomorfos ^16^.

En cuanto a la localización, el compromiso del sistema nervioso central ocurre en el 10 %, aproximadamente, de los pacientes con trasplante de órgano sólido e, incluso, puede ser el sitio primario ^5^. Otras localizaciones incluyen los ganglios linfáticos (10 a 33 %), el tubo digestivo (10 a 29 %), el hígado (5 a 12 %) y el pulmón (4 %) ^8^^,^^20^^,^^21^. Es común que los PTLD afecten el órgano trasplantado: alrededor del 15 % de los receptores de riñón presenta compromiso del injerto y esta proporción puede ser incluso mayor en el caso de los órganos cardiotorácicos. La mayoría (>90 %) de los casos de PTLD asociados con trasplante de órgano sólido tienen su origen en el receptor ^22^, en tanto que, la mayoría (>90 %) de los asociados con el trasplante de células madre hematopoyéticas se originan en el donante ^23^.

En varias series de PTLD después de trasplante de hígado, se reporta compromiso extraganglionar hasta en el 81 % de los casos ^16^, con porcentajes significativos (42 %) de invasión de órganos vecinos (estómago: 12 %, intestino delgado: 15 %), así como del mesenterio o los ganglios linfáticos retroperitoneales (40 %) ^17^. En la serie aquí descrita, la localización más frecuente correspondió al hilio hepático (extraganglionar), seguida del hígado y el bazo, en tanto que el compromiso de la médula ósea solo se detectó en un caso (cuadro 3).

Los PTLD constituyen una importante causa de morbilidad y mortalidad en los receptores de órganos sólidos en general, y de trasplante de hígado en particular. Aunque la incidencia promedio de los PTLD después de los trasplantes se ha incrementado durante los últimos años, el de hígado parece ser una excepción, probablemente por la tendencia a disminuir o, incluso, suspender el tratamiento inmunosuporesor en una significativa proporción de pacientes adultos, así como por el uso de estrategias de prevención, específicamente en los casos pediátricos ^1^^,^^5^. Los factores de riesgo clásicos para los PTLD incluyen el estado serológico del EBV en los pacientes, el órgano trasplantado y el esquema inmunosupresor, entre otros ^17^.

Hasta la fecha, este es el primer análisis clínico y patológico de los PTLD en pacientes con trasplante de hígado en Colombia. En general, las características epidemiológicas y de presentación clínica y patológica de la serie aquí descrita, son similares a las señaladas por otros autores en estudios adelantados en Latinoamérica, norteamérica e, incluso, Asia, pero se observó una notable diferencia en la frecuencia de PTLD de presentación temprana (menos de 12 meses), y se sabe que la infección por EBV, entre otros factores, se relaciona con este fenómeno. Los resultados del análisis deben motivar a la comunidad médica del país a adelantar estudios más profundos que permitan una mejor caracterización de los PTLD y de otros fenómenos en los pacientes con trasplante y que ofrezcan oportunidades para mejorar su pronóstico.
